# Chromosome 15q11-q13 Duplication Syndrome: A Review of the Literature and 14 New Cases

**DOI:** 10.3390/genes15101304

**Published:** 2024-10-08

**Authors:** Maria Bisba, Christina Malamaki, Pantelis Constantoulakis, Spiros Vittas

**Affiliations:** 1 MicroGenome, 25th Martiou 55 Str., 564 29 Thessaloniki, Greece; mpismpamaria@gmail.com; 2 Genotypos Science Labs Medical SA, 3-5 Ilision Str., 115 28 Athens, Greece; cmalamaki@genotypos.gr (C.M.); pconstantoulakis@genotypos.gr (P.C.)

**Keywords:** chromosome 15q11-q13 duplication syndrome, genetic syndromes, array-CGH, neurodevelopmental disorders, variable expressivity, reduced penetrance

## Abstract

The 15q11.2q13 chromosomal region is particularly susceptible to chromosomal rearrangements due to low-copy repeats (LCRs) located inside this area. Specific breakpoints (BP1-BP5) that lead to deletions and duplications of variable size have been identified. Additionally, this specific region contains several imprinted genes, giving rise to complex syndromes (Prader–Willi, Angelman and 15q11-q13 duplication syndromes). 15q11.2-q13 duplication syndrome has been associated with neurodevelopmental disorders (hypotonia, developmental delay, speech delay and seizures) and ASD but is characterized by variable expressivity and reduced penetrance, features that make genetic counseling a complex procedure especially in prenatal cases. In the present study, a total of 14 pre- and postnatal cases were diagnosed as 15q11.2q13 duplication carriers using Affymetrix CytoScan 750 K array-CGH, and our analysis combined these with 120 cases existing in the literature. The inheritance pattern of the cases of this study is unknown, but as a review of the literature revealed, 62.96% of the affected carriers inherited the duplicated area from their mother. The combined results of this analysis (the present study and the literature) show that in the majority of the cases, the phenotype is a compound phenotype, with clinical characteristics that include ASD, intellectual disability, developmental delay and an absence of speech. The aim of this paper is to deliver new possibilities to genetic counseling that can be provided in prenatal and postnatal cases as the phenotype of 15q11.2q13 microduplication carriers cannot be fully predicted; so, clinical diagnoses should be a combination of molecular findings and clinical manifestations that are present.

## 1. Introduction

Chromosome 15 is an acrocentric chromosome with low-copy repeats (LCRs). LCRs are located on the proximal long arm of chromosome 15 (15q11.2q13), a feature that leads to cytogenetic abnormalities such as deletions and duplications. Non-allelic homologous recombinations (NAHRs) between specific breakpoints (numbered BP1 to BP5), during meiosis I, are suspected to be the underlying mechanism through which these abnormalities are taking place [1,2].

Deletions of the 15q11.2q13 chromosomal region have been associated with two distinct neurodevelopmental syndromes: Prader–Willi syndrome (OMIM:176270), with a phenotype that includes severe hypotonia in early birth age, obesity, ASD, short stature and Angelman syndrome (OMIM:105830), the last of which is characterized by intellectual disability, seizures, microcephaly, absence of speech and a smiley face [3]. The different phenotypic features of the above syndromes have been related to the imprinted genes included in the deleted area between BPs 2 and 3 [4]. In the majority of cases, typical paternal deletions in Prader–Willi syndrome consist of two types: micro-deletion syndromes type I and type II with the deleted area extended between BPs 1 and 3 and between BPs 2 and 3, respectively [5].

Duplications of the same chromosomal region are also known. 15q11-q13 duplication syndrome (OMIM:608636) is characterized as a neurodevelopmental disorder with autosomal dominant inheritance type. Its phenotypes share some common phenotypic features with deletion syndromes such as ASD, seizures, ataxia, behavioral problems and developmental delay but can also have distinct phenotypes with dysmorphic features including an upturned nose, epicanthal folds and downslanting palpebral fissures, a characteristic EEG biomarker involving excessive beta oscillations (12–30 Hz) [6]. The duplication usually occurs in one of two forms, either the isodicentric chromosome 15q (idic(15q)) or an interstitial duplication of the critical region [7,8]. The duplication syndrome is characterized by variable expressivity with remarkable diversity in the severity of its symptoms and additionally reduced penetrance, as in many cases, the duplication is inherited through an asymptomatic or mildly affected parent. Maternally inherited duplications have been more often associated with a severe pathogenic phenotype in comparison with paternally inherited ones, even though there are recorded cases of paternal inheritance with an abnormal phenotype [9].

So far, there is plenty of research regarding the deletions of the 15q11.2q13 region as the deletion phenotype is distinct and common among its carriers. On the other hand, there is no strong evidence for the distinct phenotypic characteristics of duplication carriers. What makes diagnostic work-up, patient management and prenatal genetic counseling even more difficult is the fact that among the recorded cases, some have inherited the duplication from an unaffected parent, while others have siblings unaffected by the duplication despite having the same genotype, and in all cases, the expressivity of their phenotypes is different amongst them.

The aim of this study is to find new information on the pathogenicity and the distinct characteristic features of the duplicated 15q11.2q13 chromosomal region. For this purpose, 14 new cases (pre- and postnatal) were combined with a retrospective analysis of 120 recorded cases existing so far in the literature, aiming to find a possible correlation between phenotypes or abnormal sonographic findings and the duplicated region. Overall, this article aims to deliver new possibilities to genetic counseling that can be provided in prenatal and postnatal cases in previously affected or unaffected families.

## 2. Materials and Methods

During the literature search, 46 meta-analysis projects were extracted (Table 1), and 120 pre- and postnatal cases diagnosed as 15q11.2q13 duplication carriers were recorded. Among them, 111 cases were postnatal and 9 cases were prenatal. In parallel, during a-CGH analysis performed in our laboratory in pre- and postnatal samples with various referral reasons, 14 cases were recorded as 15q11.2q13 duplication carriers (8 postnatal and 6 prenatal). a-CGH was performed using the Affymetrix Cytogenetics Whole-Genome CytoScan 750K array platform. The results were analyzed using the Chromosome Analysis Suite Software (ChAS ver3.1 Affymetrix, Thermo Fisher Scientific, Waltham, MA, USA) according to human genome assembly GRCh37:Feb.2009 hg19.

During the interpretation of the aCGH analysis results, an individual was considered a 15q11.2q13 carrier when a duplication or triplication of the chromosomal region 15q11.2q13 that included the critical genes *NIPA1* (OMIM:608145), *NIPA2* (OMIM:608146), *CYFIP1* (OMIM:606322) and *TUBGCP5* (OMIM:608147) was found, detected between breakpoints BP1 and BP2.

## 3. Results

**Figure 1:**
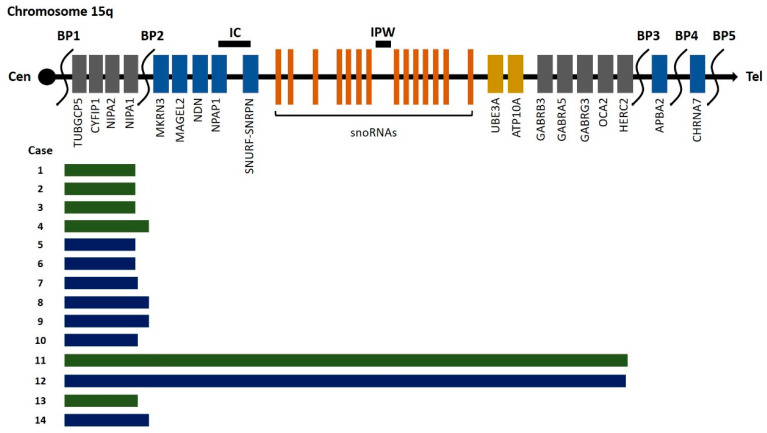
Schematic representation of the 15q11.2q13 duplication findings in the 14 cases from the present study. Prenatal cases are marked in green, while postnatal cases are marked in blue.

Out of a total of 120 cases that were recorded in the literature (Table 1), 111 of them were referred to as postnatal cases and 9 of them were referred to as prenatal cases. The phenotype of the prenatal cases could not be described, as these pregnancies were terminated because of ultrasound findings such as IUGR or heart defects (such as Fallot tetralogy) or simply because the PWS-AS chromosomal region was part of the duplication. Only one prenatal case was described as normal phenotype after birth [23]. These literature findings were combined with the results revealed from 14 new cases analyzed in our laboratory. Among them, 8 out of 14 were postnatal cases and 6 out of 14 were prenatal cases. In the majority of the new cases (12/14), the 15q11.2q13 duplication including the four critical genes, *NIPA1*, *NIPA2*, *CYFIP1* and *TUBGCP5*, was detected between breakpoints BP1 and BP2 (Figure 1).

Among the postnatal cases (Table 2), 62 out of 119 (52.10%) showed a complex phenotype with various phenotypic features shared, like intellectual disability, developmental delay, autism-related disorders, speech delay and behavioral problems. Some of them had mild dysmorphic facial features (5/119 or 4.20%), others presented with congenital heart defects (2/119 or 1.68%), and one patient showed other congenital anomalies that were not fully described (1/119 or 0.84%). One patient was referred with cerebral palsy and spastic quadriplegia (1/119 or 0.84%), but the duplication found in the following analysis was not considered responsible for his phenotype.

Regarding the prenatal cases (Table 3), the referral reason for aCGH analysis was different in each case (advanced maternal age, a family history of deafness or a positive screening test in the first trimester for T21), although two of them were diagnosed with congenital heart defects during an ultrasound examination. For all these cases, the detection of the duplication during aCGH analysis was considered a random finding, and there was no follow-up after birth.

Furthermore, concerning the 15q11.2q13 duplication copy number, the analysis did not reveal any significant difference in the expression of the phenotype between the patients that carried three or four copies of the chromosomal region between BPs 1 and 3. It is important to underline that after conventional karyotype analysis in all the new cases, the duplication carriers were carrying an interstitial duplication and not the isodicentric chromosome 15q. Additionally, according to the literature, the genes included in the duplicated area play a crucial role in the expression of the phenotype [4,9,28]. The tables below show that, in most of the cases recorded (combined results), the duplications or triplications include the critical BP1-BP2 region (Table 4 and Table 5). It is important to note that for 36 postnatal and 3 prenatal cases, there were no data concerning the size and coordinates for referral duplication.

Regarding the inheritance pattern, there are no data concerning the cases of the present study. Nevertheless, among the 111 cases extracted from the literature, 45.95% of the patients inherited the duplication from their mother. In these cases, the parent carrier did not reveal any pathogenic phenotype, which points to the low penetrance and variable expressivity of the syndrome (Table 6 and Table 7).

## 4. Discussion

The first reported 15q11.2q13 duplication case was published in 1993, and from that point, many cases with a 15q11.2q13 duplication have been recorded and studied in the literature [20]. Nevertheless, due to the similarity of phenotypic characteristics with deletion syndromes (PWS/AS) and the remarkably variable expressivity shown by the duplication syndrome, a complete phenotypic description and a phenotype/genotype correlation were never fully established. In this study, a total of 14 new cases were diagnosed (6 prenatal and 8 postnatal) carrying a 15q11.2q13 duplication, involving breakpoints BP1, BP2 and BP3 and were combined with 120 cases existing so far in the literature (9 prenatal and 111 postnatal). To our knowledge, this is by far one of the largest cohorts of 15q11.2q13 duplication subjects analyzed in a study attempting to define a more detailed phenotype/genotype correlation of this complex syndrome.

15q11.2q13 duplication syndrome is a rare congenital disease with a general population prevalence of 0.85%, affecting 1 in 30,000 to 1 in 60,000 children worldwide [27]. This duplication syndrome is characterized by ASD, seizures, ataxia, behavioral problems and developmental delay but also by hyperpigmentation and abnormal EEG, resulting in variable expressivity. In the prenatal cases, the referral reasons varied, with advanced maternal age, a positive screening test in the first trimester for T21 or congenital heart defects in some of the cases, while in postnatal cases, the probands showed a complexity of symptoms, including intellectual disability, developmental delay, autism-related disorders, speech delay and behavioral problems.

The size of the duplicated area in all the examined cases (134 in total) does not seem to play a role in the features developed (see Table 4 and Table 5). On the contrary, the presence of specific genes, the structure of the area and the pattern of inheritance appear to be more critical to the phenotypic outcome of each case. In our combined cohort, the size of the duplicated area varies from 0.43 Mbp to 9.67 Mbp, with the phenotypic characteristics being the same in all cases. Additionally, duplications seem to be more frequent than triplications (89.55%).

Regarding the inheritance pattern, in most cases the syndrome is characterized by reduced penetrance with the affected carriers of the duplicated area inheriting the duplication from a healthy parent. Although the inheritance pattern of the new cases in this study is unknown, the review of the literature (120 cases) shows that 80.20% of the carriers inherited their duplicated region from one parent (62.96% from their mother and 37.04% from their father). The additional material of maternal origin in the region is believed to be the causal factor for brain development and structure anomalies [23].

Finally, the genes included in this area play a critical role in the resulting phenotype. More specifically, four (4) genes included in the approximately 500 Kbp BP1-BP2 region (*NIPA1*, *NIPA2*, *TUBGCP5* and *CYFIP1*) are the non-imprinted genes of the area that affect the expression of neurodevelopmental characteristics, something that seems to be confirmed by the results of the present combined study [4,28]. Additionally, according to van der Zwang et al. (2010), the increased expression of *CYFIP1* and *NIPA1* genes may explain the ASD phenotype of carriers, due to their role in axogenesis and synaptogenesis [50]. *CYFIP1* overexpression in mice models led to mild learning difficulties and sensitivity to fear [51]. In the 15q11.2q13 duplication area and, more specifically, between the breakpoints BP2 and BP3, more critical genes are included such as *SNRPN* and *UBE3A* that are considered imprinted and play a critical role in the respective deletion syndromes. In parallel, in most of the cases of 15q11.2q13 duplication syndrome, the parental origin of the extra copy is maternal, a fact that demonstrates that maybe the maternally expressed imprinted genes, such as *UBE3A*, are the ones that are responsible for ASD and/or developmental disorders, as the literature underlines and the present study has found [52,53,54].

In conclusion, the present study confirms the variability of the expressed phenotype of 15q11.2q13 duplication cases. The maternal inheritance pattern in the cases that express a pathogenic phenotype is also confirmed in the majority of the cases, although penetrance of the 15q11.2q13 CNVs is difficult to estimate. The presence of the critical genes included in the BP1-BP2 region seems to play a crucial role in the pathogenicity of the resulting phenotype, while the copy number does not affect the outcome. Overall, it appears that the presence of microduplications in the chromosomal region 15q11.2q13 is a frequent finding, especially in cases with a pathological neurodevelopmental phenotype. However, diagnosis—due to the low penetrance and variable expressivity of the phenotype—should be addressed on a case-by-case basis in a combination with a molecular diagnosis and clinical outcome.

## Figures and Tables

**Table 1:** Cases recorded in the literature and used in the present study.

Study	Cases Recorded	Prenatal	Postnatal
Dawson et al., 2015 [2]	1	-	1
Urraca et al., 2013 [8]	14	-	14
Bundey et al., 1994 [10]	1	-	1
Castronovo et al., 2014 [1]	1	-	1
Schinzel et al., 1994 [11]	1	-	1
Crawford et al., 1995 [12]	1	-	1
Chadwick et al., 1996 [13]	1	-	1
Cassidy et al., 1996; Robinson et al., 1998 [14,15]	1	-	1
Long et al., 1998 [16]	1	-	1
Reddy and Logan 2000 [17]	1	-	1
Pettigrew et al., 1987; Ungaro et al., 2001 [18,19]	1	-	1
Clayton-Smith et al., 1993; Ungaro et al., 2001 [19,20]	1	-	1
Holowinski et al., 1993; Ungaro et al., 2001 [19,21]	2	-	2
Vialard et al., 2003 [22]	1	-	1
Song et al., 2022 [23]	3	3	-
Marini et al., 2013 [24]	5	-	5
Tan et al., 2014 [25]	2	-	2
Han et al., 2021 [26]	4	-	4
Huang et al., 2021 [4]	1	-	1
Ortiz-Prado et al., 2021 [27]	1	-	1
Kang et al., 2021 [9]	5	5	-
Depienne et al., 2009 [28]	3	-	3
Mohandas et al., 1999 [29]	1	-	1
Engelen et al., 1999 [30]	1	-	1
Mao et al., 2000 [31]	1	-	1
Roberts et al., 2000; Bolton et al., 2004; Veltman et al., 2005 [32,33,34]	2	-	2
Smith et al., 2004 [35]	1	-	1
Wisniewski et al., 1979 [36]	5	-	5
Al Ageeli et al., 2014 [37]	15	-	15
Browne et al., 1997 [38]	24	-	24
Baker et al., 1994 [39]	1	-	1
Cook et al., 1997 [40]	2	-	2
Repetto et al., 1998 [41]	3	-	3
Shroer et al., 1998 [42]	2	-	2
Gurrieri et al., 1999 [43]	1	-	1
Burraco et al., 2017 [44]	3	-	3
He et al., 2023 [45]	1	1	-
Ayaz-Akif et al., 2022 [46]	1	-	1
Shin Some et al., 2015 [47]	1	-	1
Basarir et al., 2023 [48]	1	-	1
Riikonen et al., 2016 [49]	2	-	2
Total	120	9	111

**Table 2:** Phenotypic features for postnatal cases (defined from both the literature and current study).

Phenotypic Features	Individuals per Feature	Percentage
Normal	15	13.04%
Developmental delay	15	13.04%
ASD	8	6.95%
Schizophrenia	1	0.88%
Epilepsy	2	1.74%
Behavioral problems	3	2.61%
Composite phenotype	62	53.91%
Congenital heart defects	2	1.74%
Other	7	6.09%
Total	115	100%

**Table 3:** Phenotypic features for prenatal cases (defined from both the literature and current study).

Phenotypic Features	Individuals per Feature	Percentage
Normal	10	71.43%
Congenital heart defects	3	21.43%
IUGR	1	7.14%
Total	14	100%

**Table 4:** Duplication size in postnatal cases (defined from both the literature and current study).

Phenotypic Features	BP1-BP2	BP1-BP3	BP2-BP3	BP2-BP4	BP2-BP5
Normal	2	3	2	-	-
Developmental delay	-	1	2	-	-
ASD	-	3	3	2	-
Schizophrenia	-	-	1	-	-
Behavioral problems	2	-	-	-	-
Composite phenotype	11	6	28	1	1
PWS-related phenotype	-	-	1	-	-
Congenital heart defects	2	-	-	-	-
Other	1	1	3	-	-
Total	18	14	40	3	1

**Table 5:** Duplication size in prenatal cases (defined from both the literature and current study).

Phenotypic Features	BP1-BP2	BP1-BP3	BP2-BP3
Normal	8	-	-
Congenital heart defects	2	1	-
IUGR	-	-	1
Total	10	1	1

**Table 6:** Pattern of inheritance (defined only in postnatal cases from the literature).

Phenotypic Features	Maternal Inheritance	Paternal Inheritance	De Novo	Unknown
Normal	2	10	-	3
Developmental delay	12	1	1	1
ASD	6	2	-	-
Schizophrenia	1	-	-	-
Epilepsy	-	1	-	1
Behavioral problems	1	1	-	-
Composite phenotype	26	13	16	3
Congenital heart defects	-	1	-	-
**Total**	**48**	**29**	**17**	**8**

**Table 7:** Pattern of inheritance (defined only in prenatal cases from the literature).

Phenotypic Features	Maternal Inheritance	Paternal Inheritance	De Novo	Unknown
Normal	1	1	3	-
IUGR	1	-	-	1
Congenital heart defects	1	-	-	-
**Total**	**3**	**1**	**3**	**1**

## Data Availability

The original contributions presented in the study are included in the article, further inquiries can be directed to the corresponding author.
